# Hairy cell leukemia in kidney transplantation: lesson from a rare disorder

**DOI:** 10.1186/2162-3619-2-22

**Published:** 2013-08-08

**Authors:** Fabrizio Vinante, Paola Tomei, Gianluigi Zaza, Alberto Zamò, Antonio Lupo

**Affiliations:** 1 Section of Hematology, Department of Medicine, University of Verona, Verona, Italy; 2 Renal Unit, Department of Medicine, University Hospital of Verona, Verona, Italy; 3 Section of Pathological Anatomy, Department of Pathology and Diagnostics, University of Verona, Verona, Italy

**Keywords:** Hairy cell leukemia, Kidney transplantation, BRAF, V600E BRAF, Immunosuppression, Calcineurin inhibitors, Post-transplant lymphoproliferative disease, Post-transplant cancer, Treatment

## Abstract

We report here on the diagnosis and successful treatment of a case of hairy cell leukemia (HCL) that arose 15 years after kidney transplantation in a 51-year-old patient. As soon as the diagnosis was made, HCL was treated with 2-CDA, obtaining complete hematological remission. Immunosuppression with the calcineurin inhibitor cyclosporin was maintained, and the graft was preserved. In kidney transplant recipients supported with immunosuppressive drugs, post-transplant lymphoproliferative diseases (PTLDs) are frequent and typically related to immunosuppression *via* a loss of control of infectious/EBV-related proliferative stimuli. To date, HCL has not been considered among PTLDs. Recently, however, the oncogenic mutation V600E of the BRAF protein kinase has been found to be a hallmark of HCL, and calcineurin inhibitors have been shown to interfere with signaling downstream of V600E BRAF early on by counteracting senescence-associated mechanisms that protect against the oncogenic potential of the mutated kinase. Such a biochemical link between the oncogene-dependent signaling and calcineurin inhibitor activities suggests that HCL in transplanted patients might be a peculiar type of PTLD based on the presence of a specific mutation. This mechanism might also be involved in other neoplasias bearing the same or similar mutations, such as melanoma and non-melanoma skin cancer.

## Background

Renal transplantation has been a major breakthrough in the treatment of end-stage renal disease, improving quality of life and reducing the mortality risk for most patients when compared with maintenance dialysis [1]. However, the rate of late graft loss remains excessive (10-year survival approximately 50%), primarily due to cardiovascular diseases and neoplasias [2,3].

Kidney transplant recipients supported with immunosuppressive drugs are approximately three times more likely to develop cancers than the general population. This excess risk is 200 and 9–20 times for Kaposi's sarcoma and nonmelanocytic/melanocytic skin cancer, respectively [4,5]. Among nonskin cancers, viral infection-related forms are predominant, such as cervical in situ disease, vulvovaginal disease, and the so-called post-transplantation lymphoproliferative diseases (PTLDs), which include Epstein-Barr virus-driven B-cell lymphoproliferation, Hodgkin's lymphoma, multiple myeloma, T/natural killer-cell lymphoproliferation, hepatosplenic γδ T-cell lymphoma and virus-induced hemophagocytic syndrome [5-7]. Hairy cell leukemia (HCL) is a rare indolent B-cell lymphoproliferative disease that induces severe, life-threatening pancytopenia and is not currently considered a PTLD. One case of HCL, which caused the patient’s death, has been reported following renal transplantation [8], and one other case has been reported in a heart transplant patient [9].

## Case presentation

A 51-year-old female was admitted to our renal unit with an 8-week history of severe neutropenia mild anemia and thrombocytopenia. She underwent a cadaveric kidney transplant in 1998 after 2 years of hemodialysis treatment for end-stage renal disease due to chronic tubular-interstitial nephritis. Following transplant, renal function was stable over 15 years with serum creatinine ranging from 106 to 133 μmol/L and no significant daily proteinuria. Maintenance immunosuppressive therapy included cyclosporin (CsA, 60+60 mg/day; C_0_=88, C_2_=488 μg/L), methylprednisolone (MP, 4 mg/day) and mycophenolate mofetil (MMF, 1 g/day). MP and MMF were discontinued due to neutropenia, but CsA was maintained.

**Figure 1:**
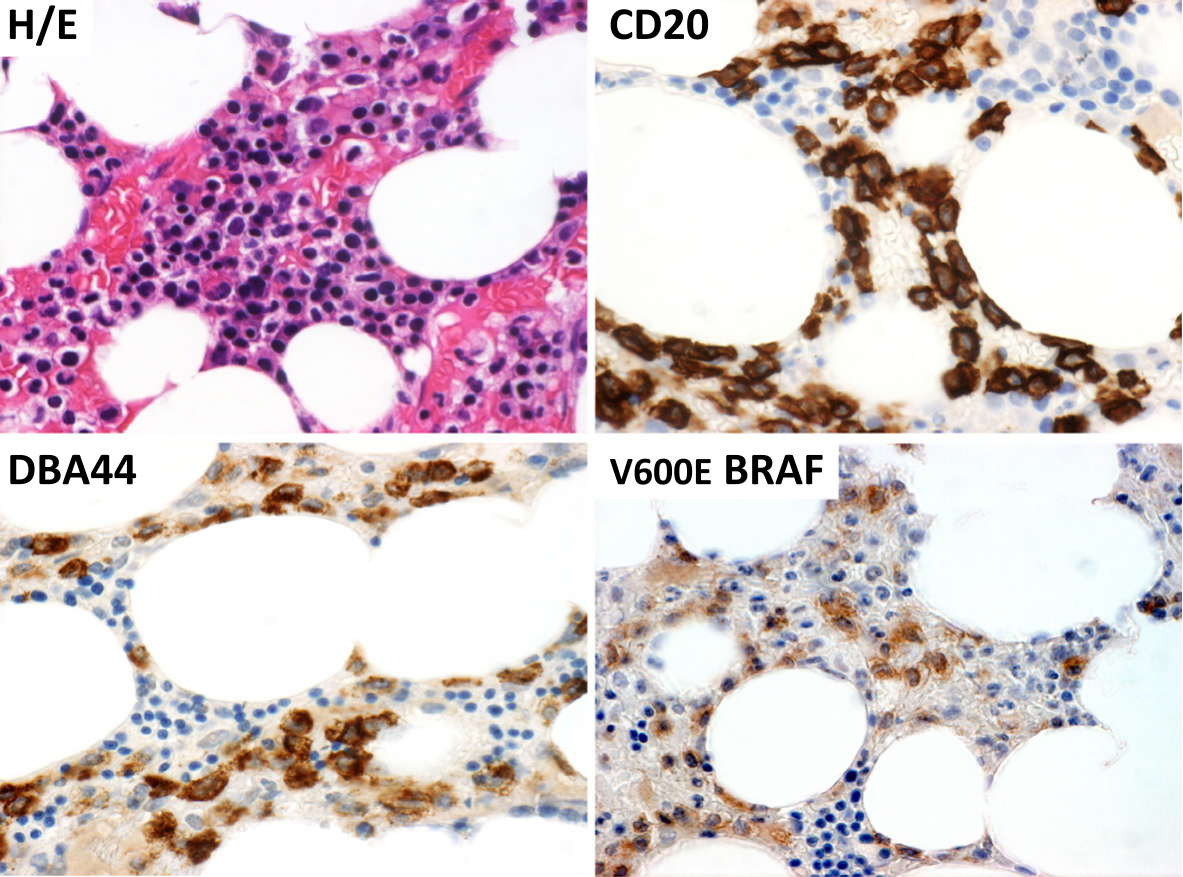
**Bone marrow biopsy.** Infiltrate of mature lymphoid cells (hematoxylin & eosin, H/E), which stained for CD20 (B cell marker), DBA44 and V600E BRAF (HCL markers). Histology and immunohistology were performed according to standard diagnostic procedures.

A physical examination, renal allograft function, abdominal ultrasonography, thoracic Rx and rheumatologic and infectious disease screening were unremarkable. Because the pancytopenia was persistent, peripheral blood and bone marrow examinations were performed. A peripheral blood smear and a flow cytometric study of circulating mononuclear cells failed to show diagnostic cells. Bone marrow could not be aspirated. Biopsy showed severely hypocellular marrow with few infiltrating B cells, which suggested lymphoproliferative disease. A contralateral biopsy showed a pattern of marrow infiltration by mature B lymphocytes that was diagnostic of HCL (Figure 1).

**Figure 2:**
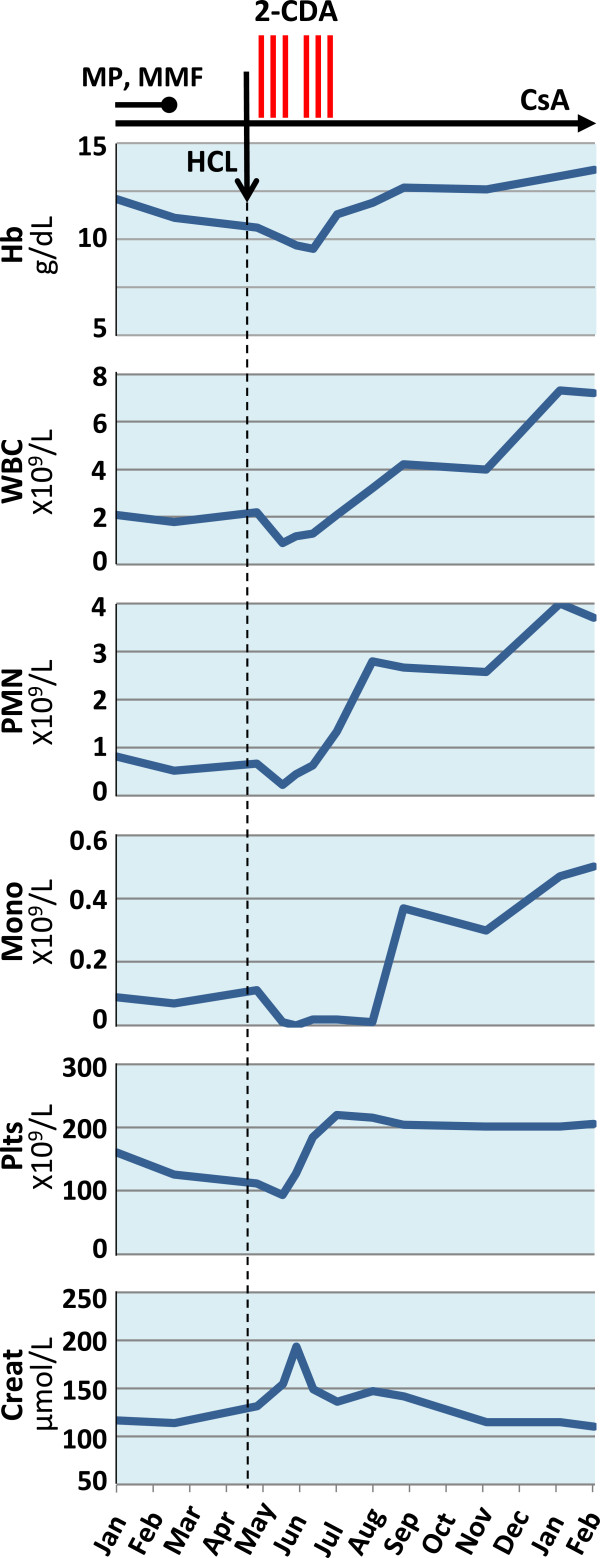
Sequential hemochrome and creatinine values in relationship to treatment.

The severely neutropenic patient was supported with both G-CSF and Epo and underwent ambulatory chemotherapy with the purine analog 2-CDA (2-Chlorodeoxyadenosine, leustatin, cladribine) according to a schedule of 0.1 mg/Kg IV once per week for 4 to 6 weeks. Prophylaxis with sulfamethoxazole-trimethoprim and recommended adequate hydration with crystalloids were performed. The 2-CDA schedule was continued for up to 6 administrations without adverse reactions or infectious complications. Due to an increase in serum creatinine to 199 μmol/L, the fourth administration was delayed one week, and the entire course of therapy lasted 7 weeks. Blood counts reached normal values approximately 2 months after starting 2-CDA. Currently, one year after stopping treatment, the patient is in continuous complete hematological remission with stable renal function (Figure 2). No bone marrow evaluation was performed.

## Discussion

Approximately 2% of leukemias in the general population are HCLs, with fewer than 2,000 new cases diagnosed annually in North America and Western Europe combined, and the disease occurs predominantly in older males [10]. After its characterization by Bouroncle and colleagues in 1958 [11], HCL was named after the “hairy” projections observed microscopically on the malignant mature B cells.

Hairy cells progressively infiltrate the bone marrow and spleen, and mild to severe pancytopenia and splenomegaly are frequent features at diagnosis. Although a full-blown leukemic picture is sometimes observed, only scant hairy cells are typically observed in the peripheral blood. High levels of soluble p55-IL-2R [12] and TNFRs [13], shed by the neoplastic cells, are detectable in the serum. TNF appears to play a pathogenic role [14] reminiscent of that in other leukemias [15,16]. Before the introduction of effective therapy, the median survival was approximately 50 months, and the natural history of HCL was one of progressive splenomegaly, bone marrow failure and death, typically due to infection. Alpha-interferon was the first treatment to obtain full remission of disease [12]. Subsequently, a variety of systemic agents have been used. 2-CDA is the preferred front-line therapy to date and is capable of inducing durable clinical remission in the majority of cases, although it fails to be curative [17,18].

Due to deep neutropenia and monocytopenia (Figure 2), HCL patients are prone to life-threatening infections, including atypical mycobacteriosis and aspergillosis. There are two relevant risks with HCL in transplant patients: life-threatening sepsis due to severe neutropenia and immunosuppression and the risk of graft loss due to either sepsis or deferred immunosuppression. Therefore, we started treatment early after diagnosis [18]. Erythropoietin and granulocyte colony stimulating factor allowed for the maintenance of adequate hemoglobin and white blood cell counts and most likely made it easier to avoid infection and complete the entire course of therapy with 2-CDA. We maintained CsA to preserve the transplant.

The pathogenesis of post-transplantation malignancies appears to be related to immunosuppression, leading to impaired immune activity against viruses, impaired immunosurveillance of neoplastic cells, DNA damage and disruption of DNA repair mechanisms and the upregulation of cytokines that can promote tumor progression (e.g., TNF, TGFβ, IL-10, VEGF). HCL is not currently considered a post-transplantation malignancy related to immunosuppression [19], but a coincidental event in a transplant patient.

Recently, however, the V600E mutation of the BRAF kinase protein (Figure 1), previously described in moles [20], melanoma and other cancers [21,22], has been recognized to be present in the majority of typical HCL cases [23]. The mutation results in the constitutive activity of BRAF kinase, which in turn triggers the Ras pathway, AP-1 and NFAT to promote cell growth [22,24]. Constitutively activated BRAF may trigger senescence, which neutralizes the mitogenic potential of BRAF [20]. Secondary events, however, may restore the full mitogenic potential of BRAF and induce lesion progression to overt cancer [20,25,26]. Calcineurin inhibitors such as CsA and tacrolimus participates in the BRAF pathway, and reduce the amount of message converging on NFAT downstream mutated BRAF [24,27-30]. By doing this, calcineurin inhibitors may counteract V600E BRAF-dependent senescence and increase the tumorigenic potential of senescent lesions [20,25,26,31].

Therefore, HCL harbors a mutated form of BRAF protein kinase [23] that results in constitutive signaling involved in oncogenesis [20,21,26-32], and calcineurin inhibitors play a part in this signaling [21,22,24-34]. This direct link between immunosuppression and a typical oncogenic feature of HCL may suggest that HCL could be considered a PTLD instead of a merely coincidental event in a host treated with calcineurin inhibitors, although it may be a peculiar PTLD entailing the V600E BRAF protein kinase mutation and most likely additional events [20,25,26,31].

Paradoxically, calcineurin inhibitors may also counteract the constitutive BRAF-driven mitogenic activity in overt cancer [25,28-31], based on the same biochemical mechanism [24,25,31,32]. Therefore, inhibitors of BRAF signaling may have the opposite effects depending on whether the V600E BRAF-bearing cells are senescent [20,25,26] or cancerous [28-30,32-34]. This duality may explain why melanomas harboring the V600E mutation of BRAF are quite frequent in transplant patients immunosuppressed with calcineurin inhibitors [4,5,21] as well as why BRAF inhibitors may have a place in melanoma treatment [21,27,30-34].

Our results suggest the following conclusions: 1) Persistent pancytopenia in a transplant patient requires timely and thorough consideration. 2) HCL should be treated as soon as possible in transplanted patients, and 2-CDA may be a good therapeutic option to induce disease remission and avoid life-threatening infection and graft loss. 3) The occurrence of a direct link between oncogenic pathways and calcineurin inhibitors in V600E BRAF-bearing cells may perhaps suggest that HCL could be included among PTLDs, although HCL might be a peculiar PTLD based on the presence of a specific mutation. 4) In general, immunosuppressive drugs may target mutation-activated oncogenic pathways to promote cancer.

## Consent

Written informed consent was obtained from the patient for publication of this case report and any accompanying images. A copy of the written consent is available for review by the Editor-in-Chief of this journal.

## Abbreviations

HCL: Hairy cell leukemia; 2-CDA: 2-Chlorodeoxyadenosine; PTLD: Post-transplant lymphoproliferative disease; CsA: Cyclosporin A; MP: Methylprednisolone; MMF: Mycophenolate mofetil; Hb: Hemoglobin; WBC: White blood cells; PMN: Neutrophils; Mono: Monocytes; Plts: Platelets; p55-IL-2R: Low-affinity receptor for interleukin 2; TNFR: Receptor for tumor necrosis factor; IL-10: Interleukin 10; TGFβ: Transforming growth factor β; VEGF: Vascular endothelial growth factor; BRAF: V-raf murine sarcoma viral oncogene homolog B encoding the serine/threonine-specific protein kinase B-Raf; V600E BRAF: BRAF mutant that leads to valine (V) being substituted for by glutamate (E) at codon 600 in the protein kinase B-Raf; Ras: Small proteins with GTPase activity that activate B-Raf; AP-1: Activator protein 1, dimeric transcription factors including Fos, Jun or ATF (activating transcription factor) subunits that bind to a common DNA site, the AP-1-binding site; NFAT: Nuclear factor of activated T-cells, collective name applied to a family of transcription factors important in immunity.

## Competing interests

The authors have no relevant conflicts of interest.

## Authors’ contributions

FV, PT wrote the manuscript. FV, PT, GZ, AL participated in clinical management and discussion. AZ carried out histology and immunohistology. All authors read, discussed and approved the manuscript.
